# The complete mitochondrial genome of *Ruspolia yunnana* (Orthoptera: Tettigoniidae: Conocephalinae)

**DOI:** 10.1080/23802359.2022.2122744

**Published:** 2022-09-15

**Authors:** Jing Liu, Xiangyi Lu, Xun Bian

**Affiliations:** Key Laboratory of Ecology of Rare and Endangered Species and Environmental Ministry of Education, Guangxi Normal University, Guilin, China

**Keywords:** *Ruspolia yunnana*, Conocephalinae, mitochondrial genome, phylogeny

## Abstract

The complete mitochondrial genome of *Ruspolia yunnana* was 15,794 bp and consisted of 37 genes and a control region. The A + T content of the mitogenome was as high as 71.3%. Most protein-coding genes started with the codon ATN and ended with stop codons TAA. Phylogenetic analyses based on mitogenomes suggested that *Ruspolia yunnana* and *Ruspolia dubia* have the closest genetic relationship. In general, this study provided meaningful genetic information for *Ruspolia yunnana* and validated the phylogenetic relationships within the Conocephalinae.

*Ruspolia yunnana* Lian & Liu, 1992 (Orthoptera: Tettigoniidae: Conocephalinae) belongs to genus *Ruspolia* Schulthess, 1898. There are about 40 recognized species recorded in this genus, which are widely distributed in the world except polar regions (Cigliano et al. 2021). Only eight species have been reported from China including: *R. dubia*, *R. interruptus*, *R. indica* and *R. lineosa* and so on (Zhou et al. 2012). In recent years, with the rapid development of sequencing technology, the amount of sequencing data of mitochondrial genomes of Orthoptera insects is increasing day by day. However, there are few studies on molecular systematics of Conocephalinae. At present, only eight mitogenomes have been sequenced, including three from *Ruspolia*. Therefore, in order to accumulate data for studying the phylogenetic relationships of Tettigonidae, the whole genome of *Ruspolia yunnana* was sequenced by high-throughput sequencing technology, and then the whole mitogenome sequence was assembled and analyzed.

Specimens of *Ruspolia yunnana* were collected in West Mountain of Kunming (25°19′12″N, 102°49′48″E), Yunnan Province, China (18 August 2019). The specimen was preserved in absolute alcohol at the Key Laboratory of Ecology of Rare and Endangered Species and Environmental Protection, Guangxi Normal University (Xun Bian and xunbian2010@163.com) under the voucher number GXNUXZ000053. Samples used in this study were approved by Animal Ethics committee for experimentation, granted by Guangxi Normal University. The total genomic DNA was extracted from muscle tissues of hind femur using the TIANamp Genomic DNA Kit (TIANGEN), and sent to Beijing Berry Genomics Co., Ltd. for high-throughput sequencing. With reference sequence of *Ruspolia dubia* (GenBank accession number: EF583824), the mitochondrial genome was assembled by NOVOPlasty 4.2 (Dierckxsens et al. 2017) and annotated using MITOS Web Server (Bernt et al. 2013).

The complete mitogenome of *Ruspolia yunnana* was 15,794 bp in length (GenBank accession number: MZ128147), including 13 protein-coding genes (PCGs), 22 transfer RNA genes (tRNAs), 2 ribosomal RNA unit genes (rRNAs) and a control region (A + T-rich regions), of which 23 genes (9 PCGs and 14 tRNAs) are located on the majority strand (J-strand) and the remaining genes (4 PCGs, 8 tRNAs and 2 rRNAs) encoded on the minority strand (N-strand). The gene order was the same as the orther insects of Conocephalinae. The overall base composition of the mitogenome was A (36.3%), T (35.0%), C (17.7%) G (10.9%) in *Ruspolia yunnana*. The mitogenome exhibited an obvious AT nucleotide bias, and it seems to be typical for orthopteran and tettigoniids. (Fenn et al. 2008 and Öztürk and Çıplak 2019) The A + T content reached 71.3%. The mitochondrial genome of *Ruspolia yunnana* consists of 14 gene spacer regions with a total length of 125 bp. The longest gene spacer was located between *rrnL* and *trnV*; There were 15 gene overlapping regions with a total length of 74 bp. The longest gene overlapping region was located between *trnL1* and *rrnL*. The total length of 13 PCGs was 11219 bp. The base composition showed positive AT bias and negative GC bias. The A + T content of 13 PCGs was 70.2% and the A + T content in the third codons (76.0%) was significantly higher than that in the first (67.9%) and second (66.9%) codons. Most PCGs started with the codon ATN (ATA, ATG or ATT), except for *COI* gene that initiated with the codon CCG. Ten PCGs ended with typical stop codons TAA, *CYTB* with TAG, and the other two genes (*COII* and *ND5*) end with T (TA) as the incomplete stop codons. The total length of 22 tRNA genes was 1332 bp, and range in size from 63 to 71 bp. All tRNA genes could be folded into the typical clover-leaf structure except for *trnS1* lacking DHU stem. The two rRNA genes were located between *trnL1* and A + T-rich regions, separated by *trnV*, with the length of 1310 and 825 bp, respectively. The length of control region was 918 bp, and the A + T content of this region was the highest in the whole mitogenome sequence at 78.5% (Figure 1).

**Figure 1.**
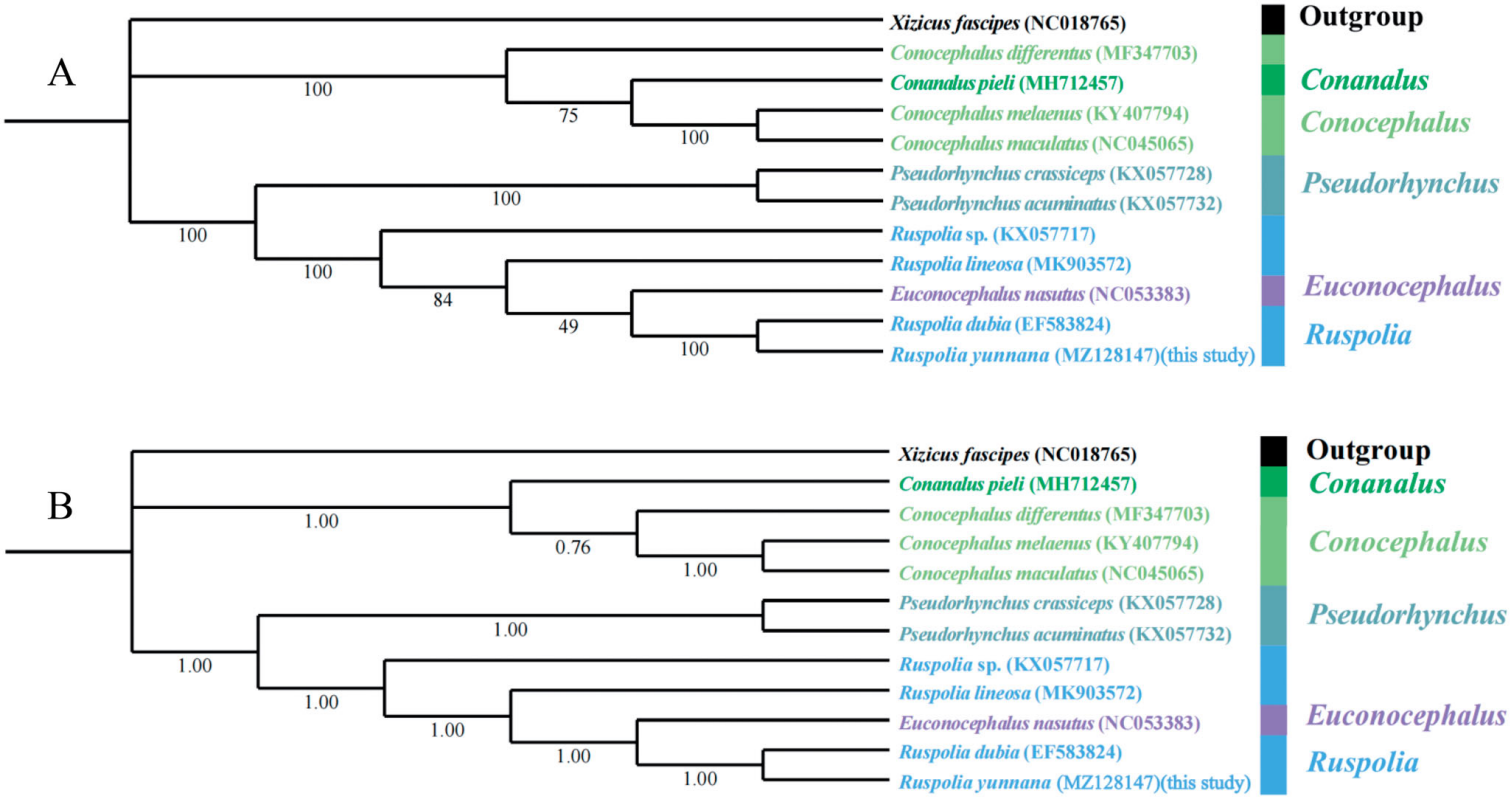
Phylogenetic trees of 11 Conocephalinae species based on 13 protein-coding genes. (A) maximum likelihood results. Applicable bootstrap values are shown. (B) Bayesian inference results. Applicable posterior probability values are shown.

The mitogenome of *Ruspolia yunnana* in this work and ten mitogenomes of Conocephalinae species from GenBank were used to perform phylogenetic analysis based on 13 PCGs, and *Xizicus fascipes* from Meconematinae was selected as outgroup. The phylogenetic trees constructed by Bayesian inference method and Maximum likelihood method using MrBayes 3.2.6 (Ronquist et al. 2012) and IQ-TREE 1.6.8 (Nguyen et al. 2015), respectively. Maximum likelihood phylogenetic trees were constructed using an ultrafast bootstrap approximation approach with 5,000 replicates. For MrBayes analyses, four simultaneous Markov chain Monte Carlo runs of 2 million generations were carried out. The trees were sampled every 1,000 generations, and the first 25% of data was discarded as burn-in. The 13 protein-coding genes were aligned with MAFFT 7.313 (Rozewicki et al. 2019) and the most suitable models for datasets were assessed by ModelFinder (Kalyaanamoorthy et al. 2017).

BI and ML trees provided similar topologies that five genera of Conocephalinae were divided to two clades with high nodal support values. Apart from the outgroup, three species of *Conocephalus* clustered with the *Conanalus* in one main clade. In the other main clade, two *Pseudorhynchus* species formed a monophyletic group and then clustered with the genera of *Euconocephalus* and *Ruspolia*. However, ML and BI tree topologies were different for internal relationships between *Conocephalus* and *Conanalus*. The ML tree suggests *Conanalus pieli* as an internal branch sister to *Conocephalus*, but the BI tree suggests *Conanalus pieli* as the most basal branch leading to *Conocephalus*. Both trees classified *Euconocephalus nasutus* of the genus *Euconocephalus* into *Ruspolia*, but the support in BI tree was stronger than that in ML tree. In this study, *Ruspolia yunnana* and *Ruspolia dubia* have the closest genetic relationship. In conclusion, the results not only enrich the mitogenome information of Conocephalinae, but also provide an important molecular basis for the phylogeny of Tettigoniidae.

## Author contributions

Designed and performed the experiments: Jing Liu, Xiangyi Lu; Morphological examination: Xun Bian, Xiangyi Lu; Analyzed the data: Jing Liu, Xiangyi Lu; Wrote the paper: Jing Liu; All authors reviewed the manuscript.

## Data Availability

The mitogenome sequence data that support the findings of this study are openly available in GenBank of NCBI at [https://www.ncbi.nlm.nih.gov] (https://www.ncbi.nlm.nih.gov/) under the accession no. MZ128147. The associated BioProject, SRA and Bio-Sample numbers are PRJNA794036, SRR18938648 and SAMN24603893 respectively.
